# The Impact of Hydrated Aluminosilicates Supplemented in Litter and Feed on Chicken Growth, Muscle Traits and Gene Expression in the Intestinal Mucosa

**DOI:** 10.3390/ani11082224

**Published:** 2021-07-28

**Authors:** Jakub Biesek, Aleksandra Dunisławska, Mirosław Banaszak, Maria Siwek, Marek Adamski

**Affiliations:** 1 Department of Animal Breeding and Nutrition, Faculty of Animal Breeding and Biology, UTP—University of Science and Technology in Bydgoszcz, Mazowiecka 28, 85-084 Bydgoszcz, Poland; miroslaw.banaszak@utp.edu.pl (M.B.); adamski@utp.edu.pl (M.A.); 2 Department of Animal Biotechnology and Genetics, UTP—University of Science and Technology in Bydgoszcz, Mazowiecka 28, 85-084 Bydgoszcz, Poland; aleksandra.dunislawska@utp.edu.pl (A.D.); siwek@utp.edu.pl (M.S.)

**Keywords:** aluminosilicates, carcass, cecum, chicken, immune status, intestinal barrier, litter, meat quality

## Abstract

**Simple Summary:**

Poultry meat production has many challenges; one of them is the optimized use of natural feed and litter additives. Aluminosilicates have many properties, stimulating both the health and growth of birds and influencing the hygienic status of production. The objectives of the study were to compare growth, meat quality traits and gene expression in the intestinal mucosa of chickens, where halloysite and zeolite were added to the feed and litter simultaneously. There was a similar growth performance in all tested groups. There was no negative impact on most of the meat characteristics, and a positive effect on the water-holding capacity of the breast muscles was observed. The immunostimulatory and immunoregulatory properties of natural minerals have been demonstrated. Therefore, their use in the production of broiler chickens can be recommended.

**Abstract:**

The aim of the study was to compare the production, muscle traits and gene expression in the intestinal mucosa of chickens supplemented with aluminosilicates in feed and litter simultaneously. A total of 300 Ross 308 were maintained for 42 days. Group 1 was the control group. In group 2, 0.650 kg/m^2^ of halloysite was added to the litter and 0.5–2% to the feed (halloysite and zeolite in a 1:1 ratio); in group 3, we added zeolite (0.650 kg/m^2^) to the litter and 0.5–2% to the feed. The production parameters, the slaughter yield and analyses of muscle quality were analyzed. There was a higher body weight, body weight gain and feed conversion ratio on day 18 and 33 in group 3, and a higher feed intake on day 19–33 in groups 2 and 3 than in 1. A lower water-holding capacity was found in the breasts of group 2 and in the legs of group 3 compared to group 1. The expression of genes related to the immune response, host defense and intestinal barrier and nutrient sensing in the intestinal tissue was analyzed. The results show a beneficial effect on the immune status of the host without an adverse effect on the expression of genes related to intestinal tightness or nutritional processes. Due to the growth, meat characteristics and the positive impact of immunostimulant and regulating properties, aluminosilicates can be suggested as a litter and feed additive in the rearing of chickens.

## 1. Introduction

Aluminosilicates, as hydrated volcanic rock soils, are represented by many types of mineral matter and have many applications as an additive to the litter and feed in poultry production [1], as well as in broad agricultural practices, i.e., as an addition to soil and as insecticides and pesticides in plant protection or hydroponic substrates [2]. The representatives of aluminosilicates that show a positive effect on many areas are halloysite and zeolite [3]. As described in the work of Eroglu et al. [2], aluminosilicates, mainly natural zeolites, have an impact on the production results of poultry and other species of farm animals, including better weight gain and a lower feed consumption ratio, as well as their use in feed improving health status. When used as a feed additive or sprinkled in loose form directly onto litter, zeolite affects the quality of bedding [4]. The quality of the litter is important for bird health and safe production, and it is mainly expressed by the ammonia (nitrogen) level and humidity, which affects the status of the soles of the feet [5]. Footpad dermatitis is a problem in the production that affects the end product and quality of the raw material (meat) and depends largely on the quality of the litter. With dry and clear litter, this problem should not occur [6]. The quality of the obtained chicken meat depends on the production results as well as the safety and hygiene status of the building [7]. The aluminosilicates can also have a positive effect on the quality of meat [8]. Mallak et al. [9] showed an improvement in the meat production and quality when using zeolite in chicken feed. Similar conclusions were reported by Safaei et al. [10] using various aluminosilicates. The halloysite has also been tested and found to be pulverized on litter to improve hygiene conditions [11].

The introduction of feed additives does not always maintain the homeostasis of the host organism and is not neutral for the intestinal microbiota of poultry. The microbiota residing in the mucosa and lumen of the gut is a key factor in the development and regulation of the immune response as well as digestion, absorption and metabolism of nutrients [12,13]. Intestinal epithelial cells, the mucosa and bacteria present in this environment and elements of the GALT system (gut-associated lymphoid tissue) together form the intestinal barrier. This is the most important barrier against external factors and the penetration of harmful microorganisms and the toxins they produce. Nevertheless, it allows the selective penetration of essential nutrients, electrolytes and water from the intestinal lumen into the circulatory system [14]. For sustainable poultry production, it is necessary to provide natural solutions which would support intestinal health and to boost chickens’ immune system without adversely affecting weight gain or the quality of the animal raw material.

The tested hypothesis is as follows: The addition of halloysite and zeolite to feed and litter at different levels affect the production results, meat quality traits, immune status and intestinal barrier condition based on gene expression in the intestinal mucosa of broiler chickens.

The aim of the study was to compare the production results and the selected breast and leg muscle traits, and evaluate the overall immune status and intestinal barrier condition based on gene expression in the intestinal mucosa of broiler chickens, which were kept with the addition of halloysite and zeolite simultaneously in the feed and litter.

## 2. Materials and Methods

The chickens were kept in the conditions similar to commercial ones (Ross 308 requirements). According to the Directive no. 2010/63/EU, the consent of the Ethics Committee was not required. According to legal requirements, the slaughter of animals is not a procedure requiring approval from the Ethics Committee, and it was performed in accordance with the applicable rules regarding the protection of animals and their welfare at the time of slaughter. In addition, it was not necessary to obtain the consent of the Ethics Committee as dictated by the Regulation 13/2016 of the National Ethical Committee for Animal Experiments.

The presented results are part of the research and development project “Safe Farm”, where the proposed use of mixtures of natural aluminosilicates was recommended and determined by experts after preliminary pilot studies [15]. The presented work is a form of application (implementation) research as a response to the needs of poultry producers.

### 2.1. Animals and Diets

In the experiment, 300 Ross 308 commercial hybrids were used. One-day-old male chicks were divided into three equal groups composed of 10 repetitions (10 birds each). Group 1 (control) was maintained without experimental factors. In group 2, halloysite (0.650 kg/m^2^) was pulverized into the litter, and in group 3 we used zeolite (0.650 kg/m^2^). A total of 187.20 kg of halloysite and 187.20 kg of zeolite was used during the whole rearing period. The litter was in the form of straw pellets, which was characterized by 97.23% of dry matter and a water-holding capacity of 314.06 kg H_2_O/100kg. Chickens were housed according to the Ross 308 (Aviagen) requirements and management. Chickens were stocked at a maximum of 42 kg/m^2^. Lighting was kept at a 24-h cycle during the first and last three days of life, with the interim period including a 6-h blackout. Light intensity was maximum 20 lx. Temperature for the first three days was on average 31 °C, followed by gradual reduction to achieve average of 20 °C from the 29th day. The humidity in the building was max. 70%. Analytical composition of the granular feed is presented in Table 1. The chemical composition and physical properties of the zeolite used are described in the work of Biesek et al. [16] (same supplier). At the same time, zeolite and halloysite were added to both experimental groups. The content of added minerals in the feed and their characterization are also presented in Table 1. Zeolite and halloysite were added to the litter in a powdery form at the five time points (1st day of rearing and four feed change). The aluminosilicates were added to the feed at source as recommended by the supplier and the project evaluation experts and mixed with the feed mill from which the feed was purchased. Access to feed and water was ad libitum. 

### 2.2. pH and Total Nitrogen in the Litter

To test the total nitrogen content and pH values of the litter, samples were collected on the day of insertion (1 day), days of the feed change (11, 19, 33 days) and on the day of slaughter (42 days). Bulk samples of pellets from each group, weighing 1 kg, were collected into sterile string bags, with five replications. Sampling was performed once on the mentioned days. The pH was determined by the potentiometric method [17,18] using the Orion 2 Star Thermo pH-meter, at the temperature of 20 °C, after calibration in standard buffers of known pH value (2.00, 4.00, 7.00, 9.00). The reading was automatic. The percentage of total nitrogen was determined according to PN-EN ISO 5983-1:2006 [19]. The determination consisted of using organic compounds of sulfuric acid in the presence of a catalyst, an alkalized solution and distillation and titration with hydrochloric acid of ammonia bound in boric acid. The FOSS Kjeltec 8400 Analyzer Unit, Sampler 8420 and FOSS Tecator Digestor apparatus were used. The analyses were performed in order to check the pH value trends in the litter and the presence of total nitrogen.

### 2.3. Growth Performance

Production parameters were recorded during the entire rearing period. Chickens were weighed on day 1 of hatching and on day 10, 18, 33 and 42 (BW, g). At the same time, the weight of the feed consumed (FI, g) was controlled. On the basis of the obtained data, body weight gain (BWG, g) was calculated for each rearing period according to the type of feed. The feed conversion ratio (FCR, kg/kg), the average daily weight gain (ADBWG, g) and then the European Broiler Index (EBI) were calculated. The status of the soles of the chickens’ feet was analyzed on the point scale to confirm the presence of footpad dermatitis according to the method described by Budnik [20].

### 2.4. Quality Traits of Breast and Leg Muscle 

From each group, 10 chickens (30 in total) were selected for slaughter and assessment of physicochemical characteristics. The selection of birds for slaughter was randomized, and 1 bird was selected from the duplicate; in total 10 chickens were selected as representing the group. Chickens were marked with individual numbers. The slaughter was performed after the chickens were stunned with an electric current followed by decapitation and quick bleeding. After slaughter, the carcasses were scalded in water at a temperature of 65 °C, then plucked in a mechanical chicken plucker and gutted. Feet were cut off at the ankle joint. After this, the carcasses were cooled at 4 °C in a cold store (Hendi, Poznań, Poland). Based on the difference in body weight and carcass weight, the slaughter yield (%) was calculated. After 24 h, the acidity of the breast muscle was measured (pH_24 h_). A pH meter (Elmetron, Zabrze, Poland) was used with a dagger electrode OSH-12-01 that was inserted into the breast muscle to a depth of 2 cm from the surface *(m. pectoralis major),* calibrated with known pH values = 4.00, 7.00, 9.00. Then, the breast muscles (m. pectoralis major and m. pectoralis minor) and the leg muscles were dissected from the carcass for further analysis and their carcass percentage was calculated. The right breast muscles and the right leg muscles were analyzed for color using a colorimeter (Konica Minolta, Tokyo, Japan) on the CIE Lab scale, where L* = saturation with color (lightness), a* = saturation with red color (redness) and b* = saturation with yellow color (yellowness). The color measurement was taken on the outer side of the tissue. The left breast muscles and the left leg muscles were ground in a meat grinder on fine meshes (Hendi, Poznań, Poland). Homogenized samples of the breast and leg muscles were used to analyze the water retention capacity. Water-holding capacity was analyzed by weighing the initial sample (M1), then placing the sample between two pieces of Whatmann blotting paper and covering it with a weight of 2 kg for 5 min. After the time had elapsed, the samples (M2) were weighed, and the percentage water loss was calculated from the difference using the formula $100-\frac{M1}{M2}\times 100\%$. Additionally, the analysis of the percentage of protein and intramuscular fat in the breast and leg muscles was analyzed using 80 g of ground tissue by FoodScan apparatus (FOSS, Hillerød, Denmark). Analyses of quality features of the breast and leg muscle were performed in 10 replications for each group.

### 2.5. Gene Expression in Intestine Mucosa

After slaughter, the cecal mucosa (n = 5 per group) was collected for gene expression analysis. On the basis of pilot studies (unpublished results) and the literature [21], it has been shown that the cecum provides the most information in this type of analysis. The cecum from each individual was cut lengthways after collection then rinsed in PBS and the mucosal layer was scraped with a glass slide. The collected tissues were fixed in RNA stabilizing buffer (RNA fix; EURx, Gdansk, Poland). Each tissue sample was homogenized in 1 mL of TRIzol reagent (MRC, Cincinnati, OH, USA) using a TissueRuptor homogenizer (Qiagen GmbH, Hilden, Germany) which provides speed of rotation of the homogenizing tip up to 35,000 rpm. Sterile plastic tips (TissueRuptor Disposable Probe, Qiagen GmbH, Hilden, Germany) were used for homogenization. The speed of tip rotation was increased gradually to ensure proper tissue homogenization for 30 s. A total of 200 µL of chloroform was added to the homogenate, shaken and centrifuged (12,000 rpm, 15 min). After centrifugation, the aqueous phase with the isolated RNA was collected. RNA was additionally purified using a Universal RNA Purification Kit (EURx, Gdansk, Poland) according to manufacturer’s instructions. The collected aqueous phase was added to the minicolumn for filtration and centrifuged for two minutes at maximum speed. A total of 70% ethyl alcohol was added to the obtained filtrate and then transferred to a spin column to bind to the silica membrane. Membrane-bound RNA was cleaned, and the membrane was washed twice with washing buffer. RNA was eluted in a volume of 50 µL of nuclease free water. Qualitative and quantitative control was performed using 2% agarose gel electrophoresis and spectrophotometer (Nanodrop 2000; Thermo Scientific, Wilmington, NC, USA). The RNA was stored at −20 °C until further analyses were performed. Isolated RNA was reverse transcribed to cDNA (ThermoScientific, Maxima First Strand cDNA Synthesis Kit for RTqPCR; Thermo Scientific, Vilnius, Lithuania) as specified by the manufacturer. The qPCR reaction was performed using the following reaction mixture: Maxima SYBR Green qPCR Master Mix (Thermo Scientific, Vilnius, Lithuania), 140 ng of cDNA, 1 μM of forward primer and 1 μM of reverse primer. Primer sequences were derived from the literature data, our previous published scientific reports or designed based on a cDNA nucleotide sequence using NCBI Primer Blast [22]. The selection of the reference genes was based on the literature [23,24] and our previous molecular analyzes [21]. Analysis included the geometric mean of two independent reference genes (*ACTB* and *G6PDH*). The primer sequences are shown in Table 2. The thermal program was carried out in a LightCycler 480 instrument II (Roche Diagnostics, Basel, Switzerland). The program consisted of initial denaturation (95 °C, 20 min) followed by 40 cycles of amplification (15 s, 95 °C), annealing (20 s, melting temperature for each pair of primers) and elongation (20 s, 72 °C). Melting curves were generated to test for the specificity of reactions at the end of the thermal cycling. Each qPCR reaction was performed in duplicate technical repetitions.

### 2.6. Statistical Analyses

#### 2.6.1. Growth Performance and Meat Traits

The numerical data on the production results and meat quality traits were statistically analyzed in the Statistica 13.0 software (Statsoft, Krakow, Poland). The mean values for each examined trait and the standard error of the mean (SEM) were calculated using one-way analysis of variance (ANOVA). For this purpose, subclass statistics were selected. Statistically significant differences (total effect) were analyzed using one-dimensional results. The significance of differences between the control group and each of the experimental groups (1 vs 2 and 1 vs 3), as well as between both experimental groups (2 vs 3) was verified by the basic statistics, choosing the Student’s *t*-test between the groups, assuming that the *p* value was <0.05. For the pH value and nitrogen content in the litter, the mean values for groups (1, 2, 3) and for individual days (1, 11, 19, 33, 42) were calculated. These values were verified by post-hoc test (*p* < 0.05).

#### 2.6.2. Relative Gene Expression and Statistical Analysis

Relative gene expression analysis was conducted separately for each experimental group by the ΔΔCt method [30] using ACTB and G6PDH as reference genes (geometric mean of cycle threshold (Ct) values) [31]. Statistical analyses were performed by comparing the Ct value of each experimental group with that of the control group by Student’s *t*-test (*p* < 0.05).

## 3. Results

### 3.1. Growth Performance 

During the rearing period, chicken losses were recorded at a level of less than 1%. Table 3 shows the production results of the chickens. The body weight (BW) was significantly higher in group 3, where zeolite was used in the litter and halloysite with zeolite (1:1) in the feed compared to the control group. The differences are shown for the 18th day of rearing (*p* = 0.044) and the 33rd day (*p* = 0.027). Weight gain (BWG) was significantly higher in group 3 in comparison to group 1 in the period from 19 to 33 days of rearing, when the grower 2 feed was administered (*p* = 0.043). When comparing the weight gain expressed as average daily values, it was found that in the first group they were significantly higher than in the group 2 (*p* = 0.005) and in the group 3 (*p* = 0.017). Feed intake (FI) of the grower 1 feed was significantly higher in group 3 compared to group 1 (*p* = 0.048), while when using the grower 2 feed (19–33 days), FI was higher in both experimental groups: 2 (*p* = 0.018) and 3 (*p* = 0.008) compared to the control group (1). On the days 19–33 (grower 2 feed), in group 3 a significantly higher feed conversion rate (FCR) was found than in group 1 (*p* = 0.002). In the remaining rearing periods, no statistically significant differences were found between the control and experimental groups in BW, BWG, FI and FCR. The European Broiler Index (EBI) calculation was performed, and no statistically significant differences were found between the groups (*p* = 0.567). However, there was a slight difference in favor of the experimental groups (2, 465; 3, 479) compared to group 1 (457). When analyzing the status of the soles of chickens’ feet, no skin lesions were found that would indicate the presence of footpad dermatitis.

### 3.2. pH and Total Nitrogen in the Litter

**Figure 1:**
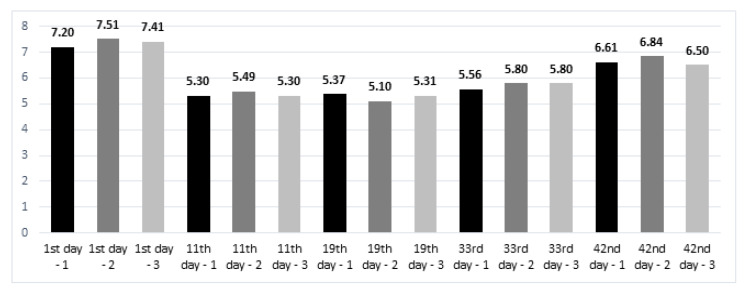
pH of the litter. Notes: 1—control group; 2—group with halloysite in the litter; 3—group with zeolite in the litter; 1st day—stater feed period; 11th day—grower 1 feed period; 19th day—grower 2 feed period; 33rd day—finisher feed period; 42nd day—slaughter day; sampling was performed once on the mentioned days. No significant differences between groups were found (*p* > 0.05); mean values from various days were significantly different, with *p*-value < 0.05.

**Figure 2:**
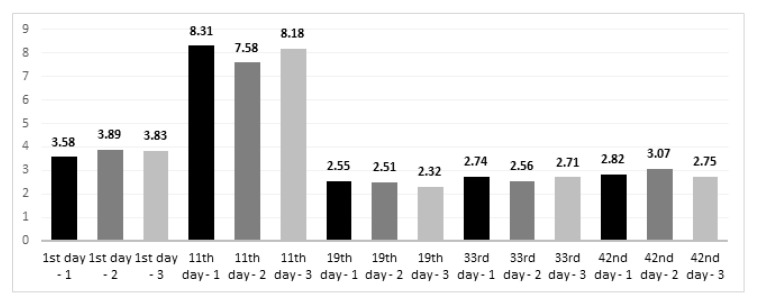
Total nitrogen (%) in the litter. Notes: 1—control group; 2—group with halloysite in the litter; 3—group with zeolite in the litter; 1st day—stater feed period; 11th day—grower 1 feed period; 19th day—grower 2 feed period; 33rd day—finisher feed period; 42nd day—slaughter day; sampling was performed once on the mentioned days. No significant differences between groups were found (*p* > 0.05); mean values from various days were significantly different, with *p*-value < 0.05; amount of nitrogen was calculated in 100 g of dry matter of the litter.

From Figure 1, showing the acidity levels (pH values) of the litter and the contents of total nitrogen (%), no statistically significant differences are observable. The acidification decreased with the first rearing period from 7.51–7.20 (alkaline pH) to 5.37–5.10 (slightly acidic pH). After the feed application step with the grower 2 type, the pH increased in all groups (6.50–6.84). Standard error of the mean (SEM) for pH of the litter was 0.22. Differences were not statistically significant between groups (*p* = 0.971 for mean values of each group at whole rearing period). The temporal changes showed that the significantly highest pH was demonstrated on the first day of rearing (7.37), compared to the other groups (*p* < 0.05). On the day of slaughter, the average pH was 6.65, with a statistically significant verification (*p* < 0.05), while the pH on day 11 (5.36) did not differ from the pH on day 19 and 33 (5.26, 5.72, respectively). A trend was noticed that in the groups with the addition of aluminosilicates, there was a quantitatively lower nitrogen content compared to the control group, and in the period from 11 to 18 days the litter contained more total nitrogen (quantitatively, 7.58–8.31%) compared to the other rearing periods. Only on day 42 was there a higher total nitrogen content in the group where the litter contained halloysite and both minerals in the feed. Standard error of the mean (SEM) was 0.56 for the nitrogen content. No significant differences between groups were found (*p* = 0.999 for mean values of each group at whole rearing period). Comparing the changes in nitrogen content in the litter during the rearing period, it was noticed that on day 1 the pH (3.77) was significantly higher than on days 19, 33 and 42 (2.46, 2.67, 2.88, respectively, *p* < 0.05). On the 11th day, the nitrogen content more than doubled (8.02), and the significance of the differences was verified at the level of *p* < 0.001. (Figure 2).

### 3.3. Slaughter Yield and Some Quality Traits of Breast and Leg Muscle 

Carcass weight from group 1 was significantly lower than in group 2 (*p* = 0.015). The slaughter yield of chickens in all groups was similar, at the level of 75.26–77.06%; however, no significant differences were found between the control and experimental groups (2, *p* = 0.057; 3, *p* = 0.298), while the one-dimensional effect of the additives used on the slaughter yield was demonstrated (*p* = 0.011). There were no significant differences in the percentage of breast muscles in the carcass between the groups, and their value was 30.97–31.75%. Similarly, no significant differences were found in the pH_24 h_ of the breast muscles (6.07–6.15) and in the color expressed on the scale of CIE L* (49.42–52.13), a* (2.25–3.18) and b* (4.64–5.37) (*p* > 0.05). Analyzing the results concerning the water-holding capacity (the value of water lost) of the breast muscles, a statistically significantly better water-holding capacity was shown in group 2 compared to group 1 (*p* = 0.012). The total effect on water absorption was found in the experiment (*p* = 0.008). The content of crude protein and intramuscular fat in the breast muscles was significantly different in all groups (*p* < 0.001). The control group showed significantly higher protein and lower fat content compared to groups 2 and 3. In group 3, the protein content was the lowest (Table 4). The share of leg muscles in the carcass was 19.48–20.59% in all groups (*p* = 0.126). A significantly higher lightness (higher L* value) was demonstrated in group 3 than that in group 1 (*p* = 0.038), while a higher yellowness (b*) was demonstrated in group 2 than that in group 1 (*p* = 0.036), and a total effect was also found yellowness in the muscles of the legs (*p* = 0.035). The water-holding capacity was lower in group 3 than in group 1 (*p* = 0.004). The protein content in the leg muscles was significantly higher in group 1 than in group 3 (*p* < 0.001), and lower than in group 2 (*p* = 0.003). On the other hand, the fat content differed between groups 1 and 2, where its content was significantly higher in the control group (*p* < 0.001). The overall effect between the groups in the chemical composition (crude protein, intramuscular fat) in the breast and leg muscles was shown to be highly significant (*p* < 0.001). Comparing the experimental groups, a significantly higher slaughter yield was shown in group 2 than in group 3 (*p* = 0.002), as well as a higher L* value and lower water loss (*p* = 0.005) and a higher protein and lower fat content (*p* < 0.001) in the breast muscles. Additionally, a higher protein and lower fat content in the leg muscles (*p* < 0.001) was found in group 2 compared to group 3. (Table 4).

### 3.4. Gene Expression

**Figure 3:**
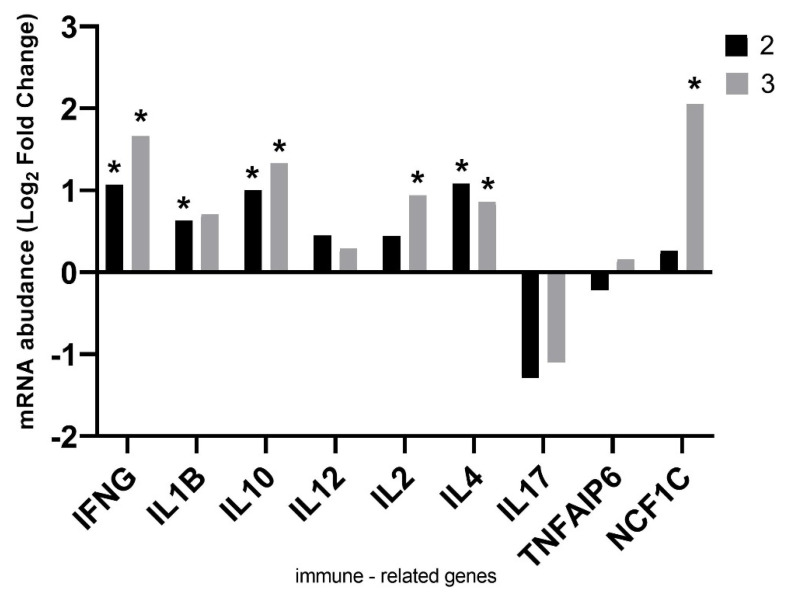
Relative gene expression of immune-related genes in cecal mucosa in chickens fed with the addition of halloysite and zeolite, as well as the addition of pulverized halloysite (group 2) and zeolite (group 3) to the litter. Statistical analysis consisted of comparing the experimental groups with the control group using Student’s *t*-test (* for *p-*value < 0.05).

The relative expression of the intestinal immune response genes analyzed in the cecal mucosa showed statistically significant upregulation of *IFNG* (*p* = 0.03 for group 2; *p* = 0.0005 for group 3) and *IL10* (*p* = 0.006 for group 2; *p* = 0.005 for group 3). The addition of zeolite (group 3) significantly increased the expression of *IL2* (*p* = 0.01) and *NCF1C (p* = 0.003) genes, and the addition of halloysite (group 2) also increased expression of *IL1β* (*p* = 0.05). The administration of both substances to litter had a numerical (*p >* 0.05) effect on the reduction of *IL17* expression. The relative expression levels of immune-related genes are presented in Figure 3.

**Figure 4:**
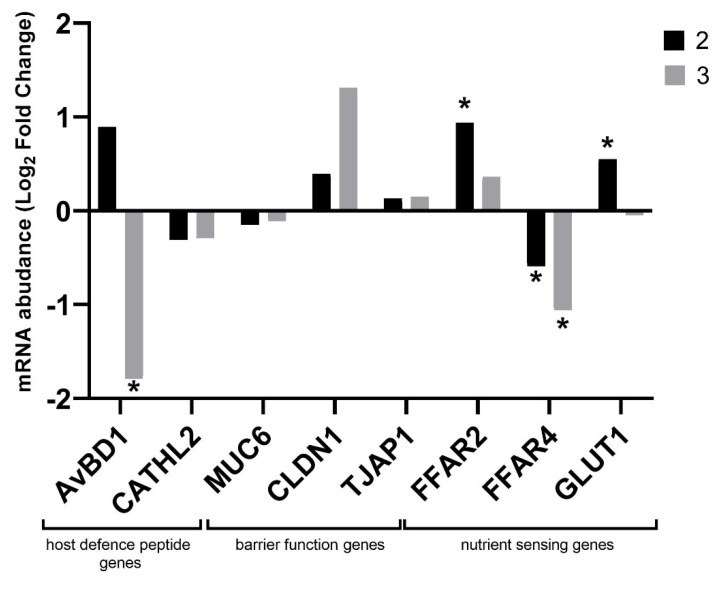
Relative gene expression of host defense peptide genes, barrier function genes and nutrient-sensing genes in cecal mucosa in chickens fed with the addition of halloysite and zeolite, as well as the addition of pulverized halloysite (group 2) and zeolite (group 3) to the litter. Statistical analysis consisted of comparing the experimental groups with the control group using Student’s *t*-test (* for *p-*value < 0.05).

The analysis of the host defense peptide genes showed a significant decrease in *AvBD1* expression (*p* = 0.03) after the addition of zeolite (group 3) to the litter. There were no significant differences in the expression of genes related to the intestinal barrier. There was a numerical increase in *CLDN1* expression after the addition of zeolite to the litter. In nutrients-sensing genes group, *FFAR2* (*p* = 0.05) and *GLUT1* (*p* = 0.005) expression increased significantly in the group with additional halloysite in the litter. There was also a significant decrease in *FFAR4* expression in both groups with added minerals (*p* = 0.02 for group 2; *p* = 0.01 for group 3). The relative expression levels of host defense genes, barrier function genes and nutrient-sensing genes are presented in Figure 4.

## 4. Discussion

### 4.1. Growth Performance and Litter Traits

Willis et al. [32] found that a zeolite addition of 2 and 3% for chickens improved their body weight gain without adversely affecting feed consumption. Our own research did not show any significant differences in body weight gain and feed consumption for the entire rearing period. Only during the feeding of grower 1 and 2 were differences in feed consumption observed; however, this did not affect the feed conversion ratio. In the studies of Bintas et al. [33], natural zeolite levels up to 0.8% were tested. These authors also showed no statistically significant differences in the production characteristics of chickens. Due to the very low mortality rate in all groups in our own research, the European Broiler Index (EBI) in all groups was considered high and amounted to over 450. Hence, the example values in the Aviagen guide in 2015 year amounted to approximately 350. The higher EBI value is very beneficial and indicates a balanced growth and good health status of birds [34].

In the level of total nitrogen in the litter, it was noticed that after 11 days of rearing, there was a visible increase in nitrogen content. At that time, there was a change of feed from starter to grower 1. According to the information in the study of Carvalho et al. [35], the nitrogen level increased after 14 days of rearing broiler chickens, which was caused by higher levels of methionine and cystine in the feed. No such relationships were found in the remaining rearing periods. The lower content of crude protein in the feed also reduced nitrogen excretion [36], which was noted in the authors’ own research. Nitrogen excretion is also related to the development and health of the digestive tracts of young chickens and the digestibility of the feeds [37,38]. Clays and aluminosilicates can stabilize gases and nitrogen in the manure (litter) [39], which is very important for maintaining appropriate hygienic conditions in production.

### 4.2. Meat Quality Traits

Less water loss was seen in group 2 in the breast muscles. In group 3, WHC was higher in the leg muscles. The water absorption of meat is related to myofibril proteins, the genotype and the post-slaughter period affect [40]. For example, in group 3, a greater loss of water in the breast muscles was combined with a lower protein content. As described by Xia et al. [40], WHC is highly positively correlated with protein concentration, and there is also a link with the gel structure of proteins. Changes related to WHC or the content of nutrients may be related to the oxidation of proteins, and Hashemi et al. [8] described that the addition of silver nanoparticles with zeolite could affect these features. The authors described the relationship of higher WHC, and lower level of protein oxidation. Analyzing the pH of the breast muscles, correct values were found in all groups at the level of 6.07–6.15. According to Van Laack et al. [41], the normal pH of breast muscles should be 5.96. Other studies have described normal values in the range 5.5–6.5, and this is associated with changes in the level of glycogen oxidation in meat [42]. The color saturation (L*) of the breast muscles in our study was 49.42–52.13. There were no significant differences between the groups, but it could be concluded that the meat was normal, while in the group 3 the meat could be considered normal with a slight deviation towards a light color. According to Pietrase et al. [43], normal breast muscles are characterized by an L* color at the level of 50–56.

### 4.3. Gene Expression 

In this study, the effect of the administration of natural minerals on the intestinal response shown in the expression of genes related to intestinal tightness, defense against pathogens, immune status and nutritional transport was also determined. To analyze the effects of supplementation in poultry science, one of the most studied sections in the gastrointestinal tract is the ceca, where most fermentation processes occur. The cecum harbors a more diverse and stable microbiome than illeum. It is therefore of great metabolic importance. Microbiota of this part of intestine shows metabolic activity ensured by intestinal bacteria (including *Ruminococcus*, *Streptococcus*, *Faecalibacterium*, *Lactobacillus*, and *Clostridium*), which support fermentation processes and produce short-chain fatty acids (SCFA) (e.g., butyrate) [44]. Chickens have paired cecals, which are habitat for similar bacterial communities. Additionally, in birds there is an organ called cecal tonsils, which is the main immune organ (the largest lymphoid organ of avian gut-associated lymphoid tissue (GALT)) [45]. The mucosa of these intestines contains clusters of lymphoid tissue [46], 1998. Taking into account the biological premises, the expression of genes in the cecal mucosa was analyzed in terms of changes in the gene expression profile related to the immune and metabolic status of the host.

#### 4.3.1. Immune-Related Genes

In this study we have shown that the addition of natural minerals gently stimulates the host’s immune system (the fold increase in expression for most genes is close to a value of 1). Excessive stimulation of the immune system may have a negative effect on production parameters. In this situation, the host organism redirects the metabolic energy intended for development and growth to maintain the immune system in a state of intense arousal. As described in Kominsky et al. [47], changes resulting from this condition can result in metabolic acidosis or reduced oxygen supply, leading to fundamental changes in tissue metabolism. Due to this fact, it is necessary to simultaneously monitor the production parameters and the levels of gene expression related to the immune system. The analysis of the expression of genes related to the immune response showed an increase in *IFNG* and *IL1β* expression. IFNG, apart from its antiviral activity, has important immunoregulatory functions [48]. IL1β is a pro-inflammatory cytokine that also shows protective functions. Intestinal IL1β is secreted into intestinal lumen and is a key mediator of intestinal inflammation. Its expression can be modulated by commensal intestinal microbiota. One mechanism by which microbiota promotes host resistance is stimulation of IL1β expression [49]. The increase in *IL1β* expression in chickens in the groups with the addition of natural minerals may be dependent on the gut microbiota. Stimulation of the intestinal mucosa towards immunomodulation has been shown, as indicated by the positive expression of the pro-inflammatory cytokine *IL12* and the anti-inflammatory *IL10* [50]. Increased expression of *IL2* and *IL4* was observed in our study. IL2 reduces the replication or pathogenicity of many viral pathogens by the mechanism of activating natural killer cells and cytotoxic T lymphocytes [51]. IL4 is considered an important cytokine in tissue repair [52] but also contributes to allergic airway inflammation [53]. Expression of IL17 was not statistically decreased, but it was numerically decreased. This is a beneficial effect of natural minerals because the upregulation of this cytokine may be associated with chronic diseases. As shown in the literature, lung injury may result from an inflammatory response mediated by IL17 [54]. Expression analysis showed a significant increase in the *NCF1C* gene after administration of the zeolite/halloysite mixture in the feed with the simultaneous administration of the zeolite in the litter. The protein encoded by the *NCF1C* gene belongs to a group of proteins that make up an enzyme complex called NADPH oxidase, which plays an important role in the immune system [55]. NADPH oxidase is primarily active in phagocytes, which catch and eliminate foreign bacteria and fungi. NADPH oxidase also regulates the activity of neutrophils, which play a key role in adjusting the inflammatory response to optimize the healing process [56]. Our analyses show a beneficial stimulation of the immune system without adversely affecting production parameters.

#### 4.3.2. Host Defense Peptide Genes

Defensins play an important role in the host’s innate defense, which includes the protection of the epithelium against pathogenic microbes and regulation of the endogenous gut microbiota. The decrease in the gene expression may lead to changes in innate immunity and modification of the intestinal microbiota. As a consequence, this may lead to an increase in the host’s susceptibility to disease [57]. In the expression analysis, we showed that in group 2, where the minerals in the feed and the halloysite were added to the litter, the expression increased numerically. In group 3, where zeolite was applied to the litter, the expression significantly decreased. However, an analysis of a panel of immune-related genes showed no disturbance in the organism’s immune status. 

#### 4.3.3. Barrier Function Genes

In order to maintain the integrity of the intestinal barrier, intestinal epithelial cells are tightly bound together by intercellular bridges. Tight connections between epithelial cells are a complex of proteins that includes, inter alia, claudin. Tight junctions ensure the tightness of the barrier between the intestinal microbiota and the host organism [58]. In this way, they protect the body against endotoxemia, which can occur, for example, during diseases. Research has shown that unsealing and impaired functioning of the intestinal barrier of the intestinal epithelium may be the main factor determining inflammation of the gastrointestinal tract or food hypersensitivity [14]. The analysis of the expression of genes related to intestinal integrity in the intestinal mucosa of chickens after the administration of natural minerals in the feed and in the litter showed no negative impact of the given minerals on the gap junctions and intestinal barrier integrity. The expression of *CLDN1* was shown to increase numerically when zeolite was added to the litter. Its increased expression leads to the tightening of intestinal epithelial cells. 

#### 4.3.4. Nutrient Sensing Genes

The cecum, from where the mucosa was collected for analysis of gene expression, plays a key role in maintaining gut health, reusing urine nitrogen and fermenting undigested nutrients [59]. It is involved in the absorption of electrolytes and water, but also allows for prolonged retention of the content. It is a specific reservoir of food content that is delivered from the ileum. Scientific reports indicate that there is a significant correlation between the composition of the microbiota of the cecum and the efficiency of harvesting energy, which suggests a strong correlation between bacteria inhabiting the cecum, including the mucosa, and production parameters [60]. 

Our research involved the analysis of three genes related to nutrient sensing: free fatty acid receptors (FFARs) and glucose transporter (GLUT). Proteins encoded by *FFAR2* and *FFAR4* genes, whose significant regulation has been demonstrated in this experiment, regulate lipids and glucose metabolism. Loss of function of FFARs, due to the fact that they regulate lipid metabolism, increases the risk of excessive fat gain [61]. Our research shows that the administration of minerals in the feed and, additionally, halloysite in the litter significantly increases *FFAR2* expression. *FFAR2* is activated by SCFA, which play a significant role in regulating the organisms’ energy homeostasis and intestinal immunity. SCFAs are produced by the intestinal microbiota. They are a kind of communicator between the microbiota and the immune system (they are responsible for maintaining balance in anti-inflammatory and pro-inflammatory reactions) [62]. Our research suggests altered activity of the intestinal microbiota after the addition of the minerals, but this aspect requires further specific analysis. The proteins encoded by the *FFAR4* genes mediate the release of incretin hormones from the epithelium into the lumen of the gut. These include GLP-1 and GIP, which are intestinal hormones that are released upon the response to food intake that inhibit gastric emptying and reduce appetite and food intake [63]. The mechanism is designed to stimulate satiety and reduce further feed intake [64]. The negative regulation of the *FFAR4* gene in the intestinal mucosa may be associated with the inhibition of the activity of incretin hormones and thus may be associated with the increase in body weight of the birds in the group where the minerals were added to the feed and litter. GLUTs are involved in the absorption of glucose through the epithelial cell membrane. Glucose uptake is important due to the fact that it acts as a fuel and an important metabolic substrate in the organism. Group 2 showed a significant increase in *GLUT1* expression, which seems to be beneficial in terms of its function. GLUT1 facilitates basal glucose uptake, which is necessary for growth and development in most cells [65].

## 5. Conclusions

The use of halloysite and zeolite in the ratio of 1:1 (0.5–2% in the feed) in the experimental groups, with the simultaneous addition of halloysite at the level of 650 g/m^2^ and zeolite (650 g/m^2^) to the straw pellet litter, had no negative effect on growth performance of broiler chickens. Some beneficial effects were detected, such as better water-holding capacity in the breast muscles of chickens kept on litter with halloysite. This parameter is important from the point of view of meat-processing technology. Additionally, the results suggested that natural minerals have immunostimulating properties (an increase in Th1 responses and pro-inflammatory cytokines) and immunoregulatory properties (an increase in Th2 responses and anti-inflammatory cytokines). On the basis of the obtained results, it can be suggested that the addition of aluminosilicates to the feed and litter allowed for the production of broiler chickens in a sustainable manner, without adversely affecting most of the tested traits and positive results of immunostimulating properties, which allows for the recommendation of natural feed and litter additives for safe poultry production. Considering the manufacturer’s market, zeolite is more available and better known, which may indicate that it is more recommended than halloysite. However, the quality of the meat and, above all, the slaughter yield indicate the use of halloysite being more suitable in the litter, as these values were more favorable. The analysis of the obtained results prompts further research into the optimal dose of natural minerals, which will allow for the improvement of production.

## Figures and Tables

**Table 1:** Analytical composition of feeds for broiler chickens (4 feeding phases) and characteristics of aluminosilicates.

Constituent [%]	Starter (0–11 Days)		Grower 1 (11–18 Days)		Grower 2 (18–33 Days)		Finisher (34–42 Days)	
	C(1) ^1^	E(2, 3) ^2^	C(1)	E(2, 3)	C(1)	E(2, 3)	C(1)	E(2, 3)
Dry matter	88.33	87.58	88.44	86.65	86.77	87.02	87.95	87.79
Crude ash	4.81	4.81	4.26	4.94	4.81	4.93	4.87	5.14
Crude protein	20.69	20.95	20.80	19.21	18.79	18.51	18.59	18.03
Crude fat	5.45	5.44	5.46	6.22	7.40	8.49	7.29	7.46
Crude fiber	2.17	3.56	2.57	3.73	3.31	3.19	3.41	3.29
Starch	39.19	39.87	39.15	39.99	39.55	38.11	40.72	40.43
Addition of zeolite and halloysite to feed [%]								
Zeolite and halloysite, 1:1 ratio	0	0.5	0	1	0	1.5	0	2
Characteristics of aluminosilicates [%]								
Zeolite				Halloysite				
Specific surface area	30–60 m^2^/g			Specific surface area	65–85 m^2^/g			
Bulk density	1.60–1.80 kg/m^3^			Bulk density	0.70–0.85 g/cm^3^			
Weight	2.20–2.44 kg/m^3^							
SiO_2_ (silicon dioxide)	71.30			Al (aluminum)	13.00			
Al_2_O_3_ (aluminum oxide)	13.10			Si (silicon)	12.00			
CaO (calcium oxide)	5.20			Ca (calcium)	0.40			
K_2_O (potassium oxide)	3.40			Mg (magnesium)	0.30			
Fe_2_O_3_ (iron (III) oxide)	1.90			Na (sodium)	0.10			
MgO (magnesium oxide)	1.20			K (potassium)	0.08			
Na_2_O (sodium oxide)	1.30			P (phosphorus)	0.30			
TiO_2_ (titanium oxide)	0.30			Fe (iron)	9.00			
Si/Al (silicon/aluminum)	5.40			Ti (titanium)	1.00			
Clinoptilolite	84.00			Mn (manganese)	0.20			
Cristobalite	8.00							
Mica clay	4.00							
Plagioclases	3.50							
Rutile	0.20							

Notes: ^1,^ C(1), control group; ^2,^ E(2, 3), experimental groups, the feed was in the form of a complete mixture (commercial), and its composition was in line with the recommendations for feeding broiler chickens. In the experimental feeds, aluminosilicates were added in the feed mill to form a homogeneous content. The results in the table are analytical. The characteristics of aluminosilicates are from the supplier. Analytical composition of feed was carried out by FoodScan apparatus.

**Table 2:** Primer sequences used in RT-qPCR reaction.

Gene	Name	Primer Sequences ^1^	References
*ACTB*	Actin beta	F: CACAGATCATGTTTGAGACCTT R: CATCACAATACCAGTGGTACG	[25]
*G6PDH*	Glucose-6-phosphate dehydrogenase	F: CGGGAACCAAATGCACTTCGT R: GGCTGCCGTAGAGGTATGGGA	[25]
*IFNG*	Interferon gamma	F: ACACTGACAAGTCAAAGCCGC R: AGTCGTTCATCGGGAGCTTG	[26]
*IL1B*	Interleukin 1 beta	F: GGAGGTTTTTGAGCCCGTC R: TCGAAGATGTCGAAGGACTG	[21]
*IL10*	Interleukin 10	F: CATGCTGCTGGGCCTGAA R: CGTCTCCTTGATCTGCTTGATG	[27]
*IL12*	Interleukin 12	F: TTGCCGAAGAGCACCAGCCG R: CGGTGTGCTCCAGGTCTTGGG	[26]
*IL2*	Interleukin 2	F: GCTTATGGAGCATCTCTATCATCA R: GGTGCACTCCTGGGTCTC	[28]
*IL4*	Interleukin 4	F: GCTCTCAGTGCCGCTGATG R: GGAAACCTCTCCCTGGATGTC	[29]
*IL17*	Interleukin 17	F: CCGTCTTCTGCTGAGAGGAGTG R: ACCGTTGTTCCGTCCCATCAC	[28]
*TNFAIP6*	Tumor necrosis factor-inducible gene 6 protein	F: CTGGCTGTCCCTGTGTGATT R: TCAGGTGCTATTGCTGCGAG	This study
*NCF1C*	Neutrophil Cytosolic Factor 1C	F: CTGTGGATGGTGTCACCGAA R: TGCCATTCTCACAGCCCTAC	This study
*AvBD1*	Avian beta-defensin 1	F: AAACCATTGTCAGCCCTGTG R: TTCCTAGAGCCTGGGAGGAT	[21]
*CATHL2*	Cathelicidin	F: AGGAGAATGGGGTCATCAGG R: GGATCTTTCTCAGGAAGCGG	[21]
*MUC6*	Mucin 6	F: TTCAACATTCAGTTCCGCCG R: TTGATGACACCGACACTCCT	[21]
*CLDN1*	Claudin 1	F: TCTTCATCATTGCAGGTCTGTC R: AACGGGTGTGAAAGGGTCAT	[21]
*TJAP1*	Tight junction-associatedprotein 1	F: AGGAAGCGATGAATCCCTGTT R: TCACTCAGATGCCAGATCCAA	[21]
*FFAR2*	Free fatty acid receptor 2	F: GCTCGACCCCTTCATCTTCT R: ACACATTGTGCCCCGAATTG	[21]
*FFAR4*	Free fatty acid receptor 4	F: AGTGTCACTGGTGAGGAGATT R: ACAGCAACAGCATAGGTCAC	[21]
*GLUT1*	Glucose transporter 1	F:AGATGACAGCTCGCCTGATG R:GTCTTCAATCACCTTCTGCGG	[21]

Notes: ^1^ F, forward primer; R, reverse primer.

**Table 3:** Growth performance of broiler chickens.

Item ^1^	Group ^2^				*p*-Value ^3^			
	1	2	3	SEM	Total	1 vs 2	1 vs 3	2 vs 3
BW (g)	mean values							
1-day old chicks	40.56	40.85	40.30	0.16	0.399	0.492	0.555	0.746
10 day	261.70	268.44	270.27	2.45	0.334	0.229	0.137	0.745
18 day	671.16 ^b^	681.95 ^ab^	695.40 ^a^	7.21	0.402	0.603	0.044	0.475
33 day	1960.85 ^b^	2048.83 ^ab^	2147.73 ^a^	34.28	0.079	0.345	0.027	0.495
42 day	3031.03	3177.40	3175.41	41.63	0.266	0.205	0.137	0.907
BWG (g)								
1–10 days	221.14	227.59	229.97	2.39	0.307	0.239	0.116	0.771
11–18 days	409.46	413.51	425.14	5.53	0.502	0.805	0.076	0.417
19–33 days	1289.69 ^b^	1366.88 ^ab^	1452.33 ^a^	30.98	0.097	0.367	0.043	0.768
34–42 days	1070.18	1128.57	1027.68	21.45	0.157	0.302	0.256	0.640
1–42 days	2990.47	3136.56	3135.12	41.70	0.267	0.206	0.138	0.910
ADBWG (g)								
1–10 days	30.80	30.94	30.76	0.24	0.954	0.772	0.956	0.771
11–18 days	92.28	92.20	91.02	0.55	0.599	0.957	0.362	0.417
19–33 days	77.47	74.89	74.01	0.90	0.270	0.284	0.149	0.640
34–42 days	122.25	110.85	112.86	2.59	0.159	0.066	0.125	0.768
1–42 days	72.64 ^a^	69.73 ^b^	69.59 ^b^	0.51	0.017	0.005	0.017	0.910
FI (g; per bird)								
1–10 days	220.55	234.70	232.80	2.75	0.069	0.057	0.076	0.448
11–18 days	545.63 ^b^	555.90 ^ab^	574.90 ^a^	6.62	0.191	0.496	0.048	0.788
19–33 days	1919.98 ^b^	2047.61 ^a^	2062.34 ^a^	36.70	0.006	0.018	0.008	0.364
34–42 days	1818.80	1829.09	1895.28	21.21	0.288	0.834	0.104	0.159
1–42 days	4613.51	5015.33	4847.54	79.19	0.112	0.078	0.106	0.478
ADFI (g; per bird)								
1–10 days	30.56	30.00	30.70	0.32	0.653	0.563	0.746	0.448
11–18 days	129.31	128.27	129.21	1.27	0.938	0.656	0.979	0.788
19–33 days	115.54	115.47	112.94	1.10	0.563	0.982	0.344	0.364
34–42 days	232.97	233.99	236.19	0.80	0.254	0.631	0.153	0.159
1–42 days	115.85	115.78	114.44	0.72	0.686	0.971	0.448	0.478
FCR (kg/kg)								
1–10 days	1.00	1.03	1.01	0.01	0.396	0.229	0.536	0.394
11–18 days	1.33	1.35	1.35	0.01	0.856	0.618	0.592	0.432
19–33 days	1.70 ^b^	1.84 ^ab^	2.01 ^a^	0.05	0.015	0.224	0.002	0.660
34–42 days	1.45	1.35	1.31	0.04	0.231	0.320	0.132	0.937
1–42 days	1.54	1.59	1.54	0.02	0.326	0.231	0.889	0.707
EBI	457	465	479	8.09	0.567	0.731	0.274	0.425

^a^,^b^: means in the same line with no common superscript differ between groups; ^1,^ BW, body weight; BWG, body weight gain; FI, feed intake; FCR, feed conversion ratio; ADBWG, average daily body weight gain; ADFI, average daily feed intake; EBI, European Broiler Index; ^2^ 1, control group; 2, halloysite group; 3, zeolite group; ^3^, SEM, standard error of the mean; *p*-value: Total, one-dimensional results; 1 vs 2, significance between control and halloysite groups; 1 vs 3, significance between control and zeolite groups; 2 vs 3, significance between halloysite and zeolite groups; 1 vs 2, 1 vs 3, 2 vs 3: *t*-test, *p*-value < 0.05.

**Table 4:** Slaughter yield and quality traits of breast and leg muscles of chickens.

Item ^1^	Group ^2^				*p*-Value ^3^			
	1	2	3	SEM	Total	1 vs 2	1 vs 3	2 vs 3
	mean values							
Live body weight (g)	2932.00	3070.80	3039.50	27.67	0.096	0.056	0.123	0.597
Carcass weight (g)	2225.93 ^b^	2365.95 ^a^	2287.57 ^ab^	22.16	0.029	0.015	0.255	0.083
Slaughter yield (%)	75.91 ^ab^	77.06 ^a^	75.27 ^b^	0.26	0.011	0.057	0.298	0.002
Breast muscle (%)	31.75	32.24	30.97	0.40	0.436	0.649	0.372	0.219
pH_24 h_	6.07	6.15	6.12	0.03	0.449	0.279	0.474	0.518
L*	51.61 ^ab^	52.13 ^a^	49.42 ^b^	0.53	0.081	0.721	0.082	0.015
a*	2.25	2.80	3.18	0.18	0.105	0.181	0.057	0.374
b*	4.64	5.37	4.75	0.31	0.608	0.422	0.878	0.418
WHC (%)	32.97 ^a^	28.58 ^b^	36.76 ^a^	1.27	0.008	0.012	0.198	0.005
Crude protein (%)	22.63 ^a^	22.25 ^b^	21.76 ^c^	0.07	<0.001	<0.001	<0.001	<0.001
Intramuscular fat (%)	2.36 ^c^	2.69 ^b^	3.09 ^a^	0.06	<0.001	<0.001	<0.001	<0.001
Leg muscle (%)	20.59	19.48	20.40	0.25	0.126	0.066	0.759	0.106
L*	45.68 ^b^	48.10 ^ab^	49.44 ^a^	0.71	0.087	0.199	0.038	0.358
a*	5.02	4.68	4.06	0.31	0.448	0.602	0.236	0.467
b*	4.49 ^a^	1.94 ^b^	3.53 ^ab^	0.47	0.035	0.036	0.999	0.055
WHC (%)	32.19	33.92	35.38	0.51	0.029	0.106	0.004	0.292
Crude protein (%)	19.03 ^b^	19.11 ^a^	18.56 ^c^	0.05	<0.001	0.003	<0.001	<0.001
Intramuscular fat (%)	7.17 ^a^	6.99 ^b^	7.14 ^a^	0.02	<0.001	<0.001	0.337	<0.001

^a^,^b^,^c^: means in the same line with no common superscript differ between groups; ^1^_,_ L*, lightness; a*, redness; b*, yellowness; WHC, water-holding capacity; ^2^ 1, control group; 2, halloysite group; 3, zeolite group; ^3^, SEM, standard error of the mean; *p*-value: Total, one-dimensional results; 1 vs 2, significance between control and halloysite groups; 1 vs 3, significance between control and zeolite groups; 2 vs 3, significance between halloysite and zeolite groups; 1 vs 2, 1 vs 3, 2 vs 3: *t*-test, *p*-value < 0.05.

## Data Availability

All the data, methods and results of the statistical analyses are reported in this paper. We remain at your disposal in the case of any questions.
